# Scientific landscape and visualization analysis of the link between adenomyosis and infertility from 2000 to 2024

**DOI:** 10.3389/fmed.2025.1488866

**Published:** 2025-02-19

**Authors:** Qiaomei Yang, Xinye Zheng, Fuchun Zhong, Li Chen, Jingxuan Hong, Xianhua Liu, Junying Jiang

**Affiliations:** ^1^College of Clinical Medicine for Obstetrics & Gynecology and Pediatrics, Fujian Medical University, Fuzhou, Fujian Province, China; ^2^Department of Gynecology, Fujian Maternity and Child Health Hospital, Fuzhou, Fujian Province, China; ^3^National Key Clinical Specialty Construction Program of China (Gynecology), Fujian Maternity and Child Health Hospital (Fujian Obstetrics and Gynecology Hospital), Fuzhou, Fujian Province, China; ^4^Department of Laboratory Medicine, Fujian Maternity and Child Health Hospital, College of Clinical Medicine for Obstetrics & Gynecology and Pediatrics, Fujian Medical University, Fuzhou, China; ^5^Department of Cardiology, Shengli Clinical Medical College of Fujian Medical University, Fuzhou, China; ^6^Department of Pathology, Fujian Maternity and Child Health Hospital, College of Clinical Medicine for Obstetrics & Gynecology and Pediatrics, Fujian Medical University, Fuzhou, China

**Keywords:** adenomyosis, infertility, association, bibliometric analysis, CiteSpace, research trends

## Abstract

**Objectives:**

Adenomyosis (AM) is a chronic disorder that significantly impacts women’s health and quality of life worldwide, particularly by causing progressive impairment in fertility. This study aimed to summarize and visualize the literature concerning AM-associated infertility using scientometric analysis.

**Methods:**

We conducted a literature search in the Web of Science^™^ Core Collection (WoSCC) database for “adenomyosis” and “infertility” as topics from 2000 to 2024. The collected data were organized in Microsoft Office Excel for further analysis. Bibliometric analyses and visualizations were performed using Origin, CiteSpace, VOSviewer, and the Bibliometrix package.

**Results:**

A total of 456 articles were published across 153 journals, reflecting a growing trend in both published and cited articles. The scholars with the highest output were Petraglia F., Chapron C., and Pellicer A., while the Fertility and Sterility were the most publications’ journal. China, the United States, and Italy ranked as the top three countries globally regarding relevant publications worldwide. The 190 keywords in the literature were divided into eight clusters primarily related to pathogenesis, adverse factors affecting pregnancy, treatment methods, diagnostic methods, disease progression, *in vitro* fertilization (IVF) management, infertility in women, and fertility management. Current hotspots in this field include investigating potential mechanisms of pathogenesis, diagnostic strategies, and improving pregnancy outcomes for patients with AM-associated infertility.

**Conclusion:**

This study highlights that infertility is the most significant and complex issue associated with AM. Although chronic disease management strategies, pharmacological treatments, and assisted reproductive technologies (ART) have improved fertility outcomes in women with AM, further clinical translational research is still warranted.

## 1 Introduction

Adenomyosis (AM) is a prevalent and chronic condition affecting reproductive-aged women. Pathologically, it is similar to endometriosis and is characterized by the benignly infiltrate of endometrial glands and stroma into the underlying myometrium, leading to progressive uterine enlargement (1, 2). The most common clinical manifestations of AM are abnormal uterine bleeding associated with anemia, chronic pelvic pain (such as dysmenorrhea and dyspareunia), infertility, and an increased risk of adverse pregnancy outcomes, all of which seriously affect the quality of women’s lives in their reproductive age (3, 4).

In recent years, the incidence of AM has risen, with a notable trend of the younger women being affected, and an increasing number of AM patients are of childbearing age with seeking fertility needs. Alarmingly, 19.5% of AM patients experience infertility (5), with over 80% of infertile patients attributed to AM and more than 30% of these individuals having previously failed assisted reproductive technology (ART) treatments (6). Moreover, female infertility and subfertility present complex challenges, accompanied by substantial economic burden and profound psychosocial effects (7), including elevated levels of anxiety and depression (8). Therefore, understanding the mechanisms through which AM impacts fertility has garnered significant scholarly attention, elucidating these pathways is critical for developing accurately targeted treatment strategies.

Despite extensive research on AM-associated infertility, there remains a scarcity articles that offer preliminary insight into its pathogenesis. The exact pathogenesis underlying AM’s impact on fertility have yet to be fully elucidated, hindering the development of targeted therapies and presenting an enormous scientific challenge for researchers. Consequently, a comprehensive big data analysis of the pathogenesis, research progress, trends, and focal points concerning AM-associated infertility is essential. This effort not only to facilitates the generation of innovative research ideas but also fosters collaborative global initiatives aimed at overcoming the identified challenges (9).

Bibliometrics, a field that qualitatively and quantitatively analyzes academic publishing, employs mathematical and statistical method to assess published works within specific disciplines (10). Recently, scientometric analysis and data visualization have emerged as valuable methodologies, extensively applied across various biomedical sciences and public health disciplines (11, 12). Compared to the traditional literature reviews, scientometrics with its visual capabilities offers advantages in quickly identifying research hotspots, critical issues, and guiding future exploration within exciting fields (13–15). For instance, Jin et al. (16) employed bibliometrics techniques to reveal gaps, traditional focal points, and potential prospects in menopausal syndrome research, clarifying future research directions for investigators. Despite the emergence of several literature reviews and meta-analyses on AM-associated infertility in the last two decades, there has been a notable lacking in scientometric studies exploring the link between AM and infertility.

To fill the apparent gap in knowledge, our study conducted bibliometrics analysis for drawing scientific knowledge maps and generating data visualization to reveal the relationship between AM and infertility by using multiple software tools. The statistical results of the keyword analysis were analyzed and summarized, which included publication year, countries and regions, institutions, authors, journals, relevant references, timeline view, and keyword co-occurrence and citation burst analysis from 2000 to 2024. This study aims to elucidate research trends and core challenges in AM-associated infertility, ultimately providing new perspectives and ideas for future investigations and attracting increased attention from scientific community.

## 2 Materials and methods

### 2.1 Data retrieval and extraction

We utilized the Web of Science Core Collection (WoSCC) of the Science Citation Index Expanded (SCIE) to retrieve and download the citation data on May 29th, 2024. The WoS database is recognized as one of the most authoritative and comprehensive citation databases, frequently employed for bibliometric studies due to its inclusion of nearly all impactful and high-quality journals, as well as its extensive data sources (17–19). Furthermore, previous studies have shown that the WoS for is more accurate than other databases literature-type labeling (13, 20). We chosen “Adenomyosis” and “Infertility” as our search terms. The retrieval formula used was as follows: [#1 was “Adenomyosis” OR “Adenomyomectomy “OR “Adenomyosis uteri” “OR” Cystic Adenomyosis” OR “Diffuse Adenomyosis” OR “Focal Adenomyosis “OR “Uterine Adenomyosis.” #2 was “Infertility” OR “Impaired fecundity “OR “Diminished semen quality” OR “Reproductive failure” OR “Fertility impairment” “Barrenness” OR “Sterility.” Final dataset was constructed as follows:: #1 AND #2]. The topical terms were restricted to the title, abstract, or keywords. The retrieval time range was from January 1st, 2000, to May 29th, 2024, with the search limited to the English languages and document types restricted to articles and reviews. A total of 456 pieces of literature were retrieved. The matching citation data were output as “Full Record and Cited References” and saved in “Plain Text” format.

### 2.2 Analysis method

Microsoft Office Excel 2019 was utilized to store and manage the relevant data. Subsequently, the pertinent data were subjected to further visualization analysis using OriginPro 2023, CiteSpace (version 6.1R6), VOSviewer (version 1.6.20), and the Bibliometrix package.^1^

Origin software was employed to analyze and map the number of annual publications, providing an intuitive understanding of the trends in the volume of research papers (21). CiteSpace was initially utilized for bibliometric analysis, encompassing country/regions, organization, category, cited journal, keyword, and reference (22). CiteSpace is a robust visualization tool that aids in identifying trends and hotspots within research fields by analyzing citation networks and exhibiting relationships between publications, including collaboration networks and keyword co-occurrence. Its broad user community, regular feature updates, cross-platform compatibility, and free accessibility make it the preferred software for bibliometric analysis (23). VOSviewer was used to optimize and visualize the scientific knowledge graph (24). Known for its versatility and user-friendly interface, VOSviewer excels in producing high-quality visualizations and offers extensive customization options. It efficiently processes large datasets, integrates seamlessly with major bibliometric databases, and includes text mining capabilities. Additionally, the software benefits from strong community support and comprehensive documentation, making it an invaluable tool for researchers (25). Bibliometrix provides a comprehensive analysis features for conducting the mapping of the co-occurrence network and clustering of keywords, enabling researchers to explore various aspects of scholarly communication (26). In all visualization networks, the size of the node represents the number of publications, the color of the node indicates different periods or clusters, and the thickness of the lines reflects the correlation’s strength.

**Figure 1:**
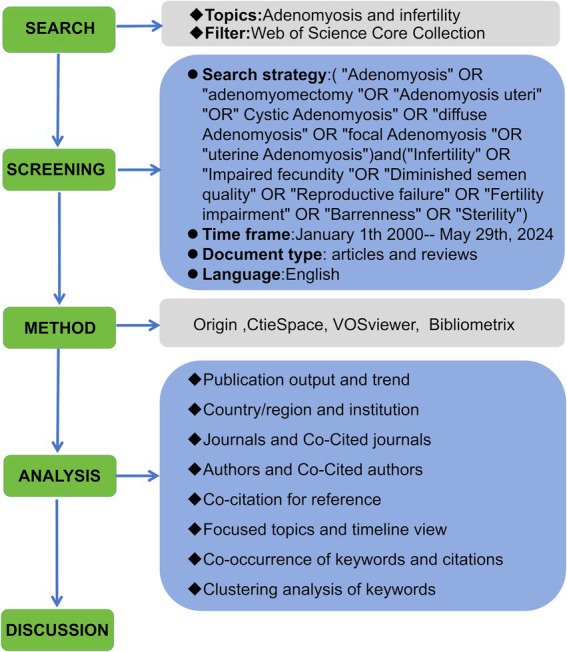
Flow chart of the scientific analysis.

The impact factor (IF) and *H*-index were included in the data table to help objectively assess the reliability and value of the journal and article research. The IF serves as a critical indicator for measuring the influence and prestige of academic journals (27), while the *H*-index evaluates scholarly contributions and predict future research accomplishments (28). To avoid bias, given that the database is updated daily, both authors individually conducted a comprehensive online search and analysis within a single day. The strategy of literature retrieval and scientometric analysis is shown in Figure 1.

## 3 Results

### 3.1 Annual publication output and trend

**Figure 2:**
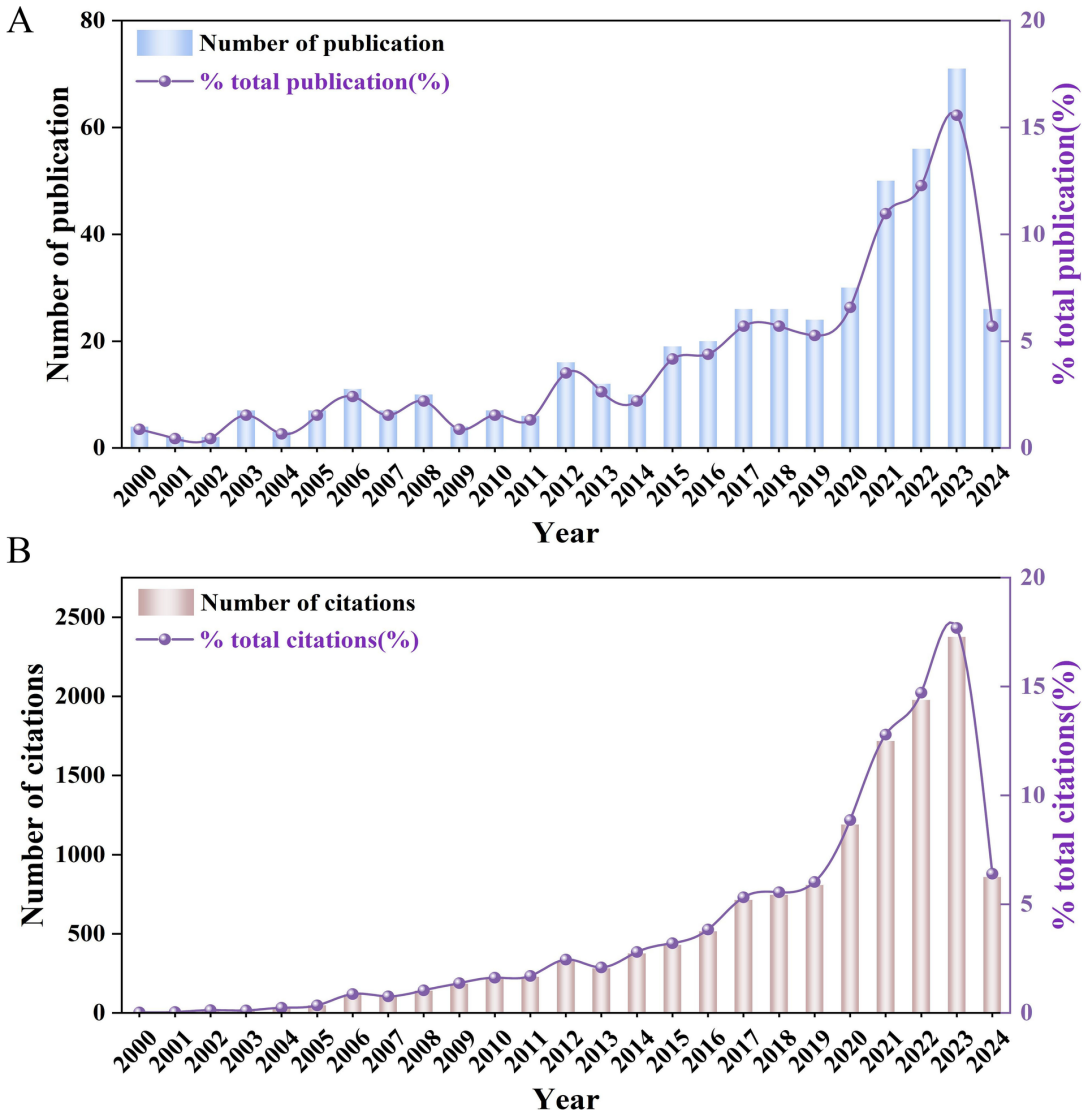
Annual publication and citation trends related to AM-associated infertility in the past 24 years. **(A)** The blue bars represent the yearly publications per year, the purple line represents the trend of annual publications in the total number of publications, and the purple solid dots represent the specific percentage (%) of yearly publications to total publications. **(B)** The brown bars represent the annual citations per year, the purple line represents the trend of yearly citations in the total number of citations, and the purple solid dots represent the specific percentage (%) of annual citations to total citations.

A total of 456 articles on AM-associated infertility from 2000 to 2024 were identified. The annual publication count is exhibited in Figure 2A. Despite some fluctuations in annual publications, the overall trend has been upward. Before 2011, published documents were primarily in the single digits, from 2012 onward, publications consistently remained in double digits. The peak occurred in 2023, with 71 publications. Numerous publishers contributed literature across various subject categories, with the top 10 publishers and categories listed in Table 1. The largest publishers are Elsevier (130); the most common research category is obstetrics and gynecology (265). Figure 2B shows the annual citation counts, totaling 13,426 citations across retrieved articles, resulting in an average number of 29.44 citations per paper. The *H*-index for screened publications was 62, indicating a steady upward trend in annual citations. In 2023, citations peaked at 2,375. Notably, the most substantial research output and citation frequency increase occurred between 2019 to 2023.

**Table 1:** Top 10 publishers and categories related to adenomyosis associated infertility in the past 24 years.

Rank	Publishers	Counts	Counts	Rank	Category	Counts	Counts
1	Elsevier	130	28.51%	1	Obstetrics Gynecology	265	58.11%
2	Springer Nature	57	12.50%	2	Reproductive Biology	177	38.82%
3	Oxford University Press	45	9.87%	3	Medicine General Internal	43	9.43%
4	Wiley	37	8.11%	4	Radiology Nuclear Medicine Medical Imaging	41	8.99%
5	MDPI	32	7.02%	5	Endocrinology Metabolism	26	5.70%
6	Taylor & Francis	25	5.48%	6	Medicine Research Experimental	18	3.95%
7	Frontiers Media SA	17	3.73%	7	Multidisciplinary Sciences	10	2.19%
8	Lippincott Williams & Wilkins	16	3.51%	8	Biochemistry Molecular Biology	9	1.97%
9	Thieme Medical Publishers	11	2.41%	9	Oncology	9	1.97%
10	Hindawi Publishing Group	6	1.32%	10	Public Environmental Occupational Health	9	1.97%

### 3.2 Distribution of countries/regions and institutions

**Figure 3:**
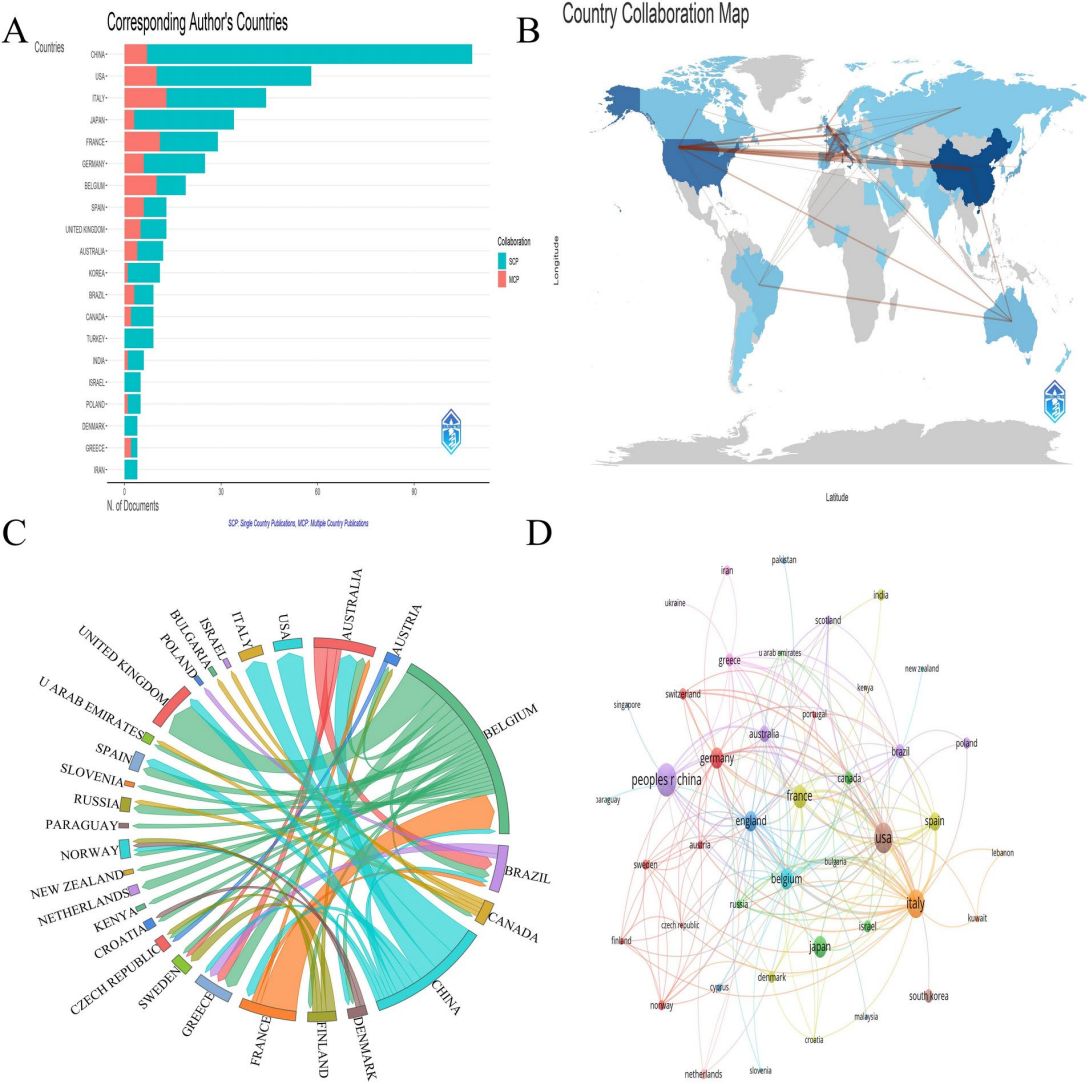
The most productive countries/regions related to AM with infertility in the past 24 years. **(A)** The top-ranking countries/areas in the articles published by the corresponding author. The orange bars represent the corresponding author’s country (MCP), and the green bars represent the second corresponding author’s (SCP). **(B)** Global distribution of the production countries/regions of the articles. **(C)** The closest cooperation network among the most productive countries/regions. **(D)** The closest cooperation network among the most productive countries/regions.

**Figure 4:**
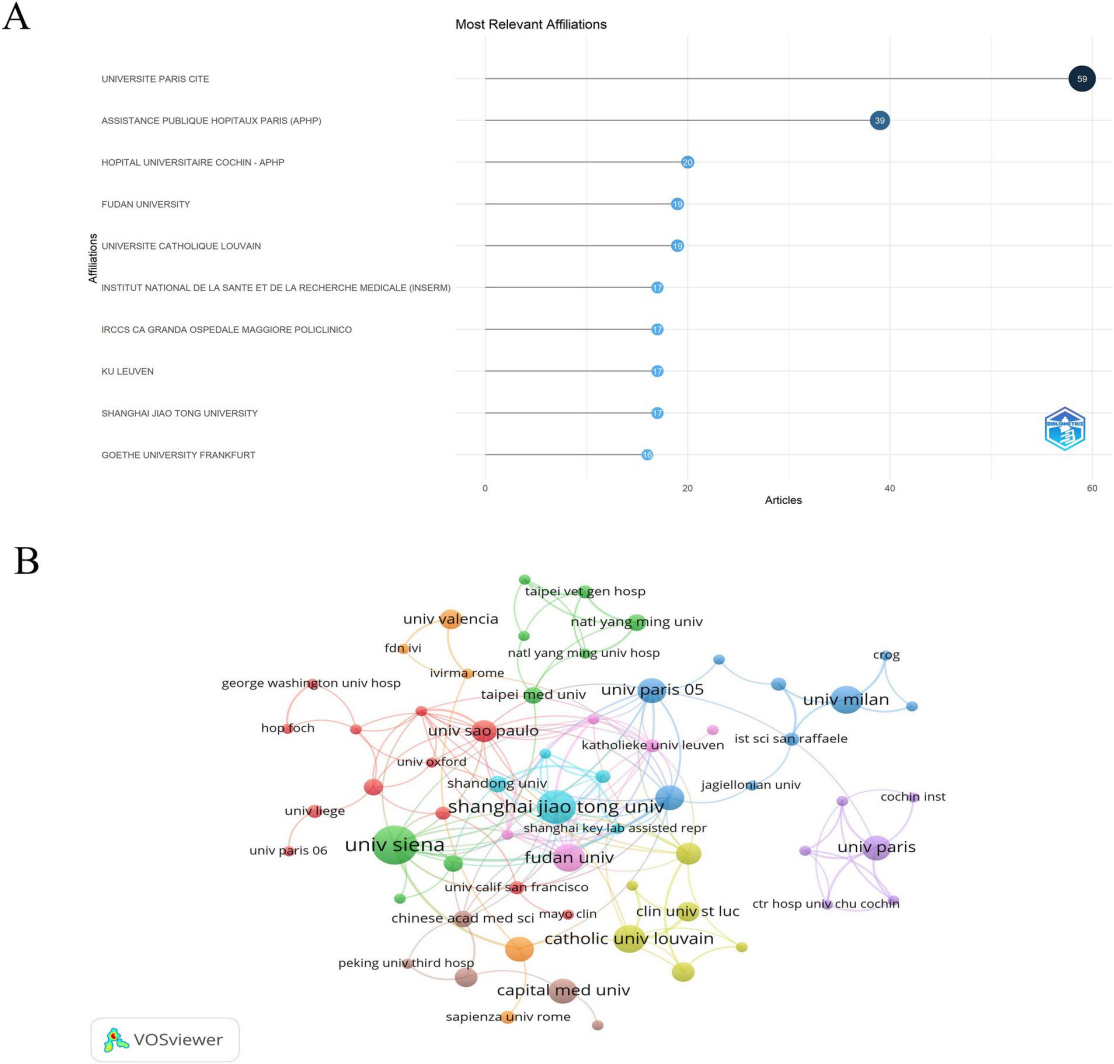
The most productive institutions related to AM with infertility in the past 24 years. **(A)** The most productive institutions. The solid blue dots represent the number of publications. **(B)** The closest cooperation network is among the most productive institutions.

The publications involved 51 countries/regions and 123 institutions. The top 10 countries/regions by total published papers are shown in Table 2. China led with 25.44% (116 articles), followed by the USA (17.54%, 80 articles), Italy (14.69%, 67 articles), France (8.77%, 40 articles), and Japan (7.46%, 34 articles). Figures 3A,B depict the top-ranking countries regarding published articles and corresponding authors, revealing China’s significant influence in AM-associated infertility research. The *H*-index for the top 10 most productive countries/regions indicates that the USA (3,051), England (1,928), Germany (1,690), France (1,514), Australia (1,377), and China (1,333) have made notable contributions. High-yield institutions mainly originate from Europe. The cooperation network analysis among countries is illustrated in Figures 3C,D, showing that China, Belgium, and France collaborated closely. The top 10 productive institutions and their cooperation network are displayed in Figure 4. Leading organizations include the Assisting the Paris Public Hospital (4.83%, 22 papers), University Paris (3.73%, 17 papers), Cochin University Hospital (3.29%, 15 papers), National Institute of Health and Medical Research (3.07%, 14 papers) and Katholieke Universiteit Leuven (3.07%, 14 papers). Additionally, institutions with prominent cooperation networks include Siena University, Shanghai Jiao Tong University, Ku Leuven Catholic University, Fudan University, and the University of Milan.

**Table 2:** Top 10 countries and organizations related to adenomyosis associated infertility in the past 24 years.

Rank	Country	Counts	Counts	*H*-index	Rank	Organizations	Counts	Counts
1	China	116	25.44%	1,333	1	Assistance Publique Hopitaux Paris APHP	22	4.83%
2	USA	80	17.54%	3,051	2	Universite Paris Cite	17	3.73%
3	Italy	67	14.69%	1,333	3	Hopital Universitaire Cochin APHP	15	3.29%
4	France	40	8.77%	1,514	4	Institut National de la Sante et de la Recherche Medicale INSERM	14	3.07%
5	Japan	34	7.46%	1,301	5	KU Leuven	14	3.07%
6	Belgium	31	6.80%	1,067	6	University of Siena	14	3.07%
7	Germany	31	6.80%	1,690	7	Sapienza University Rome	11	2.41%
8	England	29	6.36%	1,928	8	Shanghai Jiao Tong University	11	2.41%
9	Spain	22	4.83%	1,215	9	Fudan University	10	2.19%
10	Australia	17	3.73%	1,377	10	IRCCS CA Granda Ospedale Maggiore Policlinico	10	2.19%

### 3.3 Journals and co-cited journals

**Figure 5:**
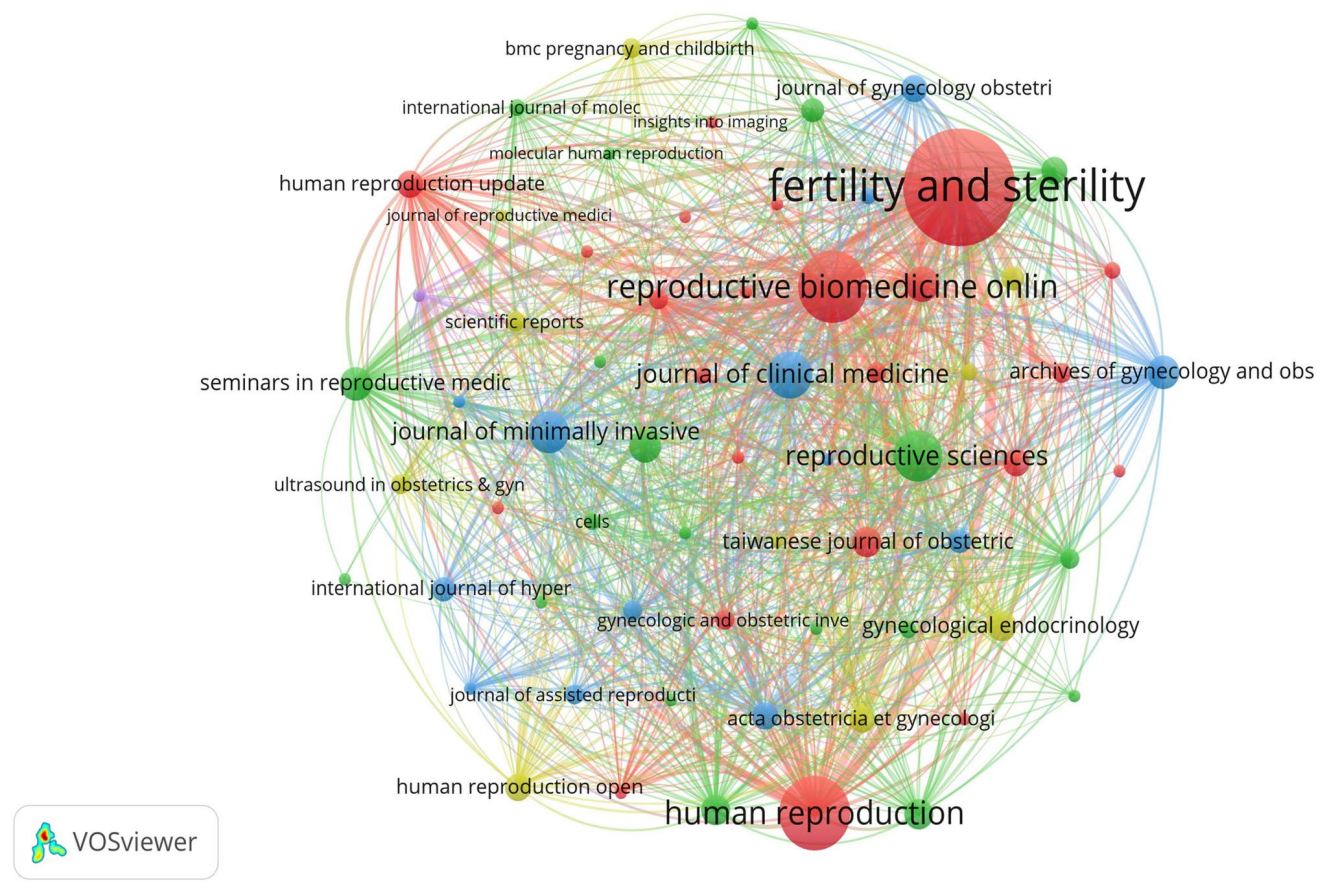
Network for the co-cited journals related to AM-associated infertility in the past 24 years. Fertility and Sterility, Human Reproduction, and Reproductive Biomedicine Online were the most co-cited journals. The node color represents the different co-cited journals, and the node size represents the number of co-cited journals. Lines of other colors show that the two keywords appear in an article. The lines between nodes represent the cross-reference relationships between different journals.

This analysis include 153 journals and 401 co-cited journals. The top 20 most productive and co-cited journals are summarized in Table 3. Fertility and Sterility (10.53%, 48 papers) published the most papers in this field, followed by Human Reproduction (5.48%, 25 papers), Reproductive Biomedicine online (5.26%, 25 papers), Reproductive Sciences (3.29%, 15 papers), American Journal of Obstetrics and Gynecology (2.85%, 13 papers) and Journal of Minimally Invasive Gynecology (2.41%, 11 papers). Co-citation network analysis is displayed in Figure 5, revealing that Fertility and Sterility was the most frequently co-cited journal, with 1,578 total citations, followed by Human Reproduction (1,477 citations), Reproductive Biomedicine Online (954 citations); Human Reproduction Update (865citations) and Best Practice & Research Clinical Obstetrics & Gynecology (441 citations). Among the top 20 journals, Human Reproduction Update had the highest IF of 13.3 in 2024, while Human Reproduction boasted the highest *H*-index of 209 in 2024.

**Table 3:** Top 20 output and most co-cited journals related to adenomyosis associated infertility in the past 24 years.

Rank	Journal	Counts	Counts	Rank	Co-cited journals	Citation counts	IF (2024)	*H*-index (2024)
1	Fertility and Serility	48	10.53%	1	Fertility and Sterility	3,080	6.9	190
2	Human Reproduction	25	5.48%	2	Human Reproduction	1,477	6.1	209
3	Reproductive Biomedicine Online	24	5.26%	3	Reproductive Biomedicine online	954	4	100
4	Reproductive Sciences	15	3.29%	4	Human Reproduction update	865	13.3	158
5	Journal of Clinical Medicine	13	2.85%	5	Best Practice & Research Clinical Obstetrics & Gynaecology	441	5.5	72
6	Journal of Minimally Invasive Gynecology	11	2.41%	6	Radiographics	297	5.5	151
7	European Journal of Obstetrics & Gynecology and Reproductive Biology	9	1.97%	7	Journal of Minimally Invasive Gynecology	267	4.1	70
8	Archives of Obstetrics and Gynecology	8	1.75%	8	Reproductive Sciences	265	2.9	70
9	Journal of Obstetrics and Gynaecology Research	8	1.75%	9	European Journal of Obstetrics & Gynecology and Reproductive Biology	240	2.6	90
10	Seminars in Reproductive Medicine	8	1.75%	10	Seminars in Reproductive Medicine	219	2.7	69
11	Frontiers in Endocrinology	7	1.54%	11	American Journal of Obstetrics and Gynecology	178	9.1	203
12	Gynecological Endocrinology	7	1.54%	12	Taiwanese Journal of Obstetrics & Gynecology	164	2.1	29
13	Reproductive Biology and Endocrinology	7	1.54%	13	Ultrasound in Obstetrics & Gynecology	162	7.1	128
14	Taiwanese Journal of Obstetrics & Gynecology	7	1.54%	14	Reproductive Biology and Endocrinology	158	4.4	79
15	Acta Obstetricia et Gynecologica Scandinavica	6	1.32%	15	Gynecological Endocrinology	155	2	53
16	Best Practice & Research Clinical Obstetrics & Gynaecology	6	1.32%	16	Journal of Obstetrics and Gynaecology research	151	1.6	44
17	Current Opinion in Obstetrics Gynecology	6	1.32%	17	Acta Obstetricia et Gynecologica Scandinavica	136	4.3	93
18	Frontiers in Medicine	6	1.32%	18	Biomed Research International	128	0	94
19	Human Reproduction Open	6	1.32%	19	Current Opinion in Obstetrics & Gynecology	118	2.1	66
20	Human Reproduction Update	6	1.32%	20	Cells	111	6	14

### 3.4 Authors and co-cited authors

**Figure 6:**
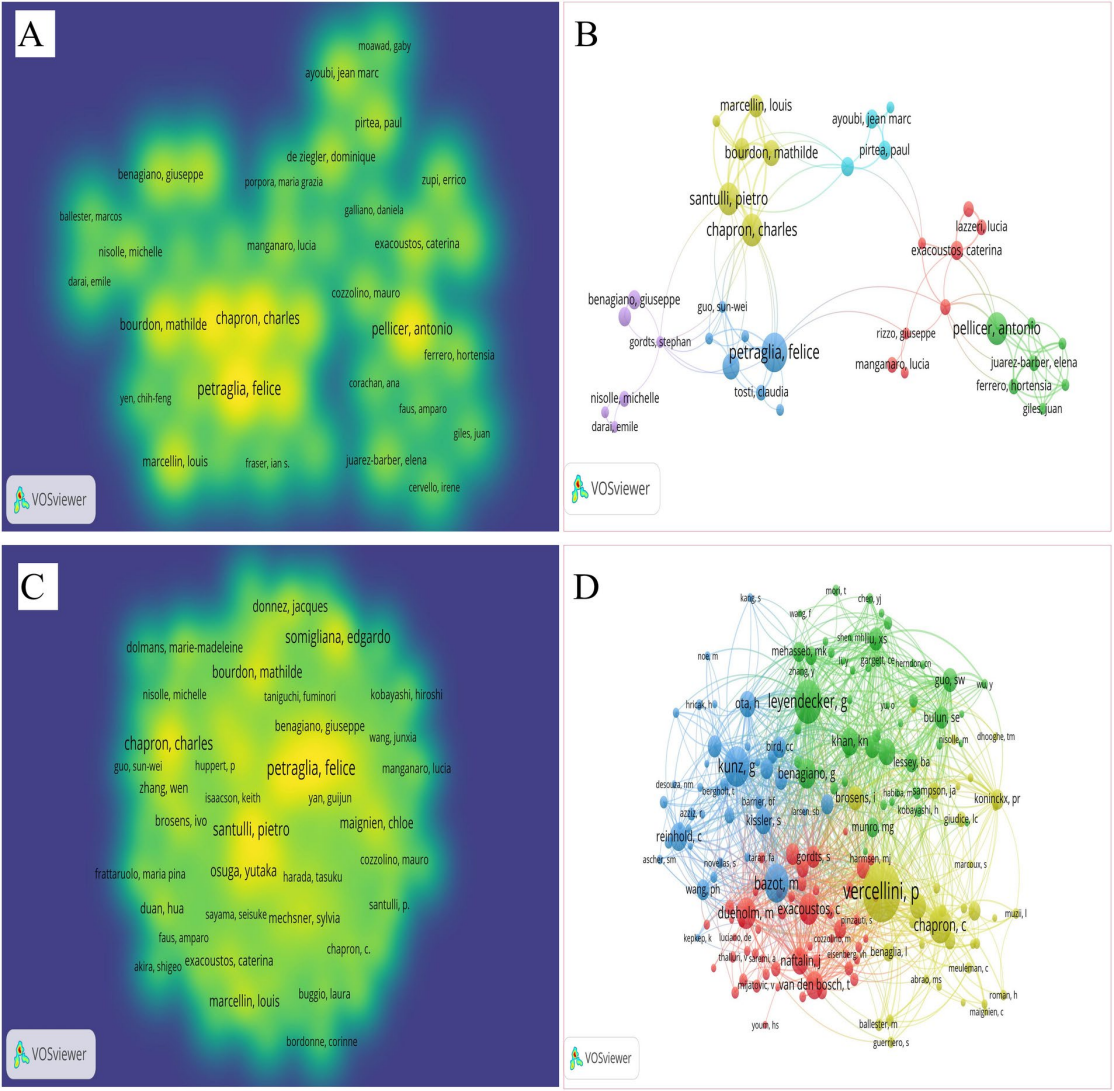
Map of authors and co-cited authors related to AM-associated infertility in the past 24 years. **(A)** Spectrum density diagram of the most productive authors. **(B)** The most productive contributing authors of the network diagram. **(C)** The spectrum density map of the co-cited authors. The authors’ closest relationship is allocated to one cluster with the same color in this cluster density map. **(D)** The cooperation of different authors with co-cited authors in the network diagram.

The analysis identified 98 authors (with more than two articles were published) and 179 co-cited authors (with over 30 citations). Figures 6A,B feature the top most productive authors and contributors, while Figures 6C,D illustrate the top cited authors and the cooperation network between different authors. Detailed information on the top 10 authors and co-cited authors is presented in Table 4. Petraglia F., Santulli P., and Pellicer A. were the most published authors, each contributing 13 papers in the field of AM-associated infertility. Following them, Santulli P. and Bourdon M. each published 10 papers. The centrality of the top 10 published authors ranged from 0.018 to 0.029, with Petraglia F., Santulli P., and Pellicer A. achieving the highest centrality of 0.029. In terms of total co-cited frequency, the leading authors were Vercellini P. (314 citations), Leyendecker G. (199 citations), Kunz G. (178 citations). The close collaboration among different authors and co-cited authors indicate their crucial role in advancing the field.

**Table 4:** Top 10 authors and co-cited authors related to adenomyosis associated infertility in the past 24 years.

Rank	Published author	Counts	Centrality	RANK	Co-cited author	Citation
1	Petraglia F.	13	0.029	1	Vercellini P.	314
2	Chapron C.	13	0.029	2	Leyendecker G.	199
3	Pellicer A.	13	0.029	3	Kunz G.	178
4	Santulli P.	10	0.022	4	Bazot M.	165
5	Bourdon M.	10	0.022	5	Chapron C.	151
6	Vannuccini S.	9	0.020	6	Dueholm M.	125
7	Maignien C.	9	0.020	7	Exacoustos C.	123
8	Marcellin L.	9	0.020	8	Benagiano G.	115
9	Ayoubi J.	8	0.018	9	Khan K.	105
10	Benagiano G.	8	0.018	10	Reinhold C.	104

### 3.5 Co-citation analysis for reference, focused topics, and timeline views

**Figure 7:**
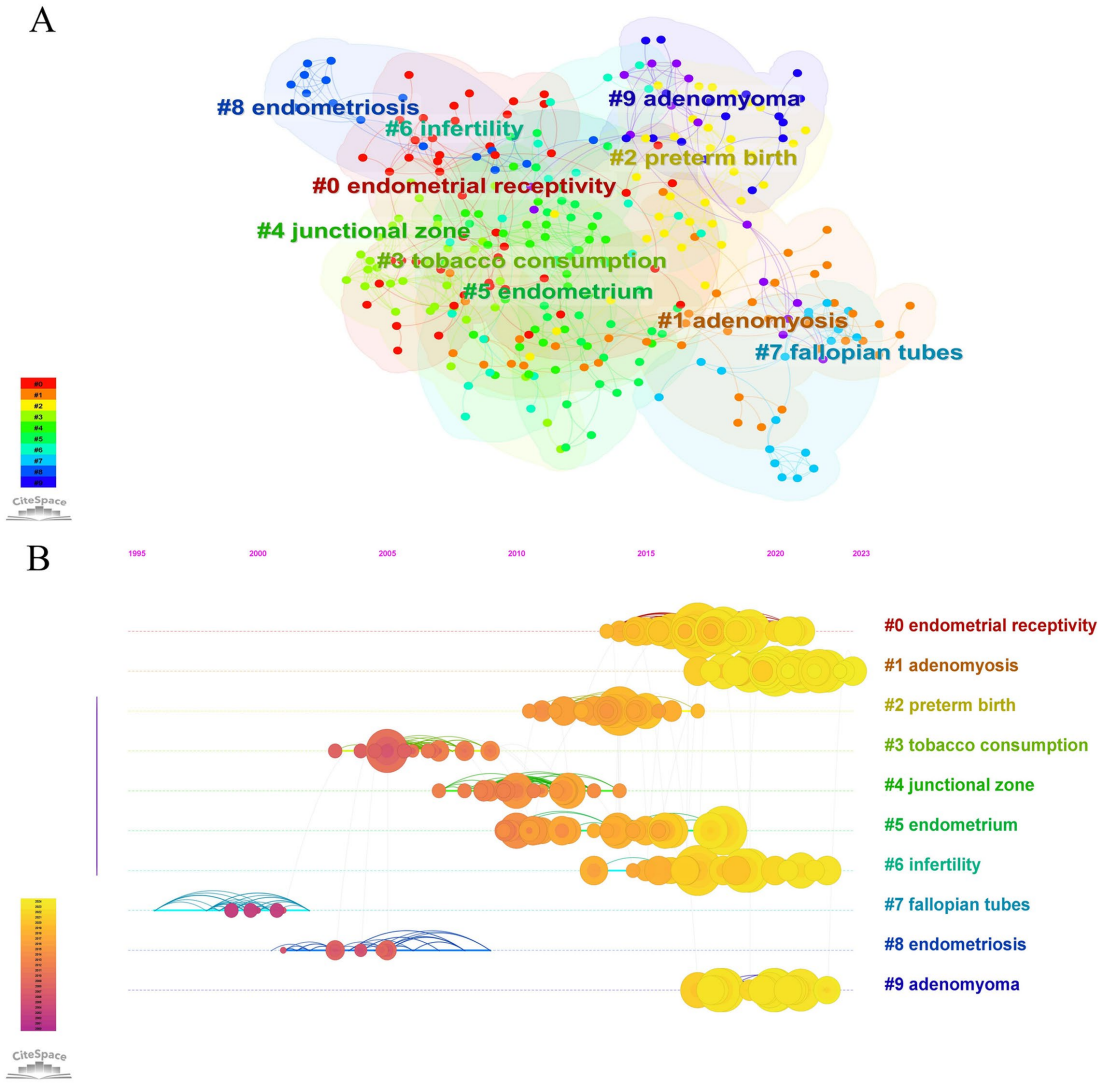
Visualization network and timeline view of co-cited papers related to AM-associated infertility in the past 24 years. **(A)** The co-citation visualization network of co-cited references. Each node delegates a review or article, and each frame delegates a cluster. The size of each node represents the number of coreferences. The tags of the clusters also showed nearly the same frames. **(B)** The timeline view of co-cited references. The position of the nodes on the horizontal axis indicates the time when the reference debuted, and the size of the nodes is positively correlated with the number of paper citations. The lines between the nodes represent cocited relationships. This blue color indicates nearly 2000, while a darker yellow color indicates almost 2024.

Table 5 lists the 30 most highly cited literature in the field of AM-associated infertility, highlighting six studies that have been co-cited over 200 times. The most co-cited article by Chen C. et al. (2017), published in Nature Communications, with 458 citations. This is followed by Koninckx P. R. et al. (2012) in Fertility and Sterility, with 325 citations, Kunz G. et al. (2005) and Vercellini P. et al. (2014) in Human Reproduction, with 276 and 242 citations, respectively. Co-citation analysis of the research topics was performed using CiteSpace, the results of which are presented in Figure 7. This analysis categorized all included papers into 10 clusters based on their primary research topics, endometrial receptivity (#0), AM (#1), preterm birth (#2), tobacco consumption (#3), junctional zone (#4), endometrium (#5), infertility (#6), fallopian tubes (#7), endometriosis (#8), and adenomyoma (# 9). Timeline view analysis indicates that the most popular research topics are endometrial receptivity (#0), AM (#1), endometrium (#5), infertility (#6), and adenomyoma (# 9). The earliest papers with citation bursts emerged between 2010 and 2015.

**Table 5:** Top 30 most highly cited literature related to adenomyosis associated infertility in the past 24 years.

Rank	Title	Author	Source title	Publication year	Total citations	Average per year
1	The microbiota continuum along the female reproductive tract and its relation to uterine-related diseases	Jia H.	Nature Communications	2017	458	57.25
2	Deep endometriosis: definition, diagnosis, and treatment	Donnez J.	Fertility and Sterility	2012	325	25
3	Adenomyosis in endometriosis—prevalence and impact on fertility. Evidence from magnetic resonance imaging	Leyendecker G.	Human Reproduction	2005	276	13.8
4	Uterine adenomyosis and *in vitro* fertilization outcome: a systematic review and meta-analysis	Somigliana E.	Human Reproduction	2014	242	22
5	Infertility and reproductive disorders: impact of hormonal and inflammatory mechanisms on pregnancy outcome	Petraglia F.	Human reproduction update	2016	216	24
6	Pathogenesis of endometriosis: the genetic/epigenetic theory	Martin D. C.	Fertility and Sterility	2019	209	34.83
7	Adenomyosis: epidemiological factors	Fedele L.	Best Practice & Research Clinical Obstetrics & Gynecology	2006	192	10.11
8	Magnetic resonance imaging and transvaginal ultrasonography for the diagnosis of adenomyosis	Olesen F.	Fertility and Sterility	2001	189	7.88
9	Pathogenesis of uterine adenomyosis: invagination or metaplasia?	Dolmans M. M.	Fertility and Sterility	2018	187	26.71
10	Oxidative stress may be a piece in the endometriosis puzzle	Mikolajczyk M.	Fertility and Sterility	2003	185	8.41
11	Diagnosing adenomyosis: an integrated clinical and imaging approach	Petraglia F.	Human Reproduction Update	2020	184	36.8
12	Pathogenesis of adenomyosis: an update on molecular mechanisms	Petraglia F.	Human Reproduction Online	2017	172	21.5
13	Uterine adenomyosis in the infertility clinic	Timmerman D.	Human Reproduction Update	2003	148	6.73
14	The impact of adenomyosis on women’s fertility	Taniguchi F.	Obstetrical & Gynaecological Survey	2016	147	16.33
15	Effects of adenomyosis on *in vitro* fertilization treatment outcomes: a meta-analysis	Tulandi T.	Fertility and Sterility	2017	146	18.25
16	Recurrence of ovarian endometrioma after laparoscopic excision	Taketani Y.	Human Reproduction	2006	146	7.68
17	Adenomyosis and subfertility: a systematic review of prevalence, diagnosis, treatment and fertility outcomes	Bhattacharya S.	Human Reproduction Update	2012	145	11.15
18	Role of medical therapy in the management of uterine adenomyosis	Petraglia F.	Fertility and Sterility	2018	142	20.29
19	Medical and surgical management of adenomyosis	Brosens I.	Best practice & Research Clinical Obstetrics & Gynaecology	2006	135	7.11
20	The role of *HOX* genes in female reproductive tract development, adult function, and fertility	Taylor H. S.	Cold Spring Harbor Perspectives in Medicine	2016	134	14.89
21	The pathophysiology of uterine adenomyosis: an update	Brosens I.	Fertility and Sterility	2012	134	10.31
22	Structural abnormalities of the uterine wall in women with endometriosis and infertility visualized by vaginal sonography and magnetic resonance imaging	Leyendecker G.	Human Reproduction	2000	132	5.28
23	MR Imaging of endometriosis: ten imaging pearls	Edward R.	Radiographics	2012	129	9.92
24	Uterine adenomyosis: a need for uniform terminology and consensus classification	Brosens I.	Human Reproduction Online	2008	125	7.35
25	Transvaginal sonographic features of diffuse adenomyosis in 18–30-year-old nulligravid women without endometriosis: association with symptoms	Petraglia F.	Ultrasound in Obstetrics & Gynecology	2015	123	12.3
26	Uterine polyps, adenomyosis, leiomyomas, and endometrial receptivity	Munro M. G.	Fertility and Sterility	2019	122	20.33
27	Uterine peristaltic activity and the development of endometriosis	Wildt L.	Uterus human Reproduction	2004	120	5.71
28	Adenomyosis and infertility	Benagiano G.	Reproductive Biomedicine Online	2012	115	8.85
29	The motile and invasive capacity of human endometrial stromal cells: implications for normal and impaired reproductive function	Gellersen B.	Human Reproduction Update	2013	113	9.42
30	Long-term pituitary downregulation before frozen embryo transfer could improve pregnancy outcomes in women with adenomyosis	Feng Y.	Gynaecological Endocrinology	2013	104	8.67

### 3.6 Analysis of co-occurrence of keywords and citations

**Figure 8:**
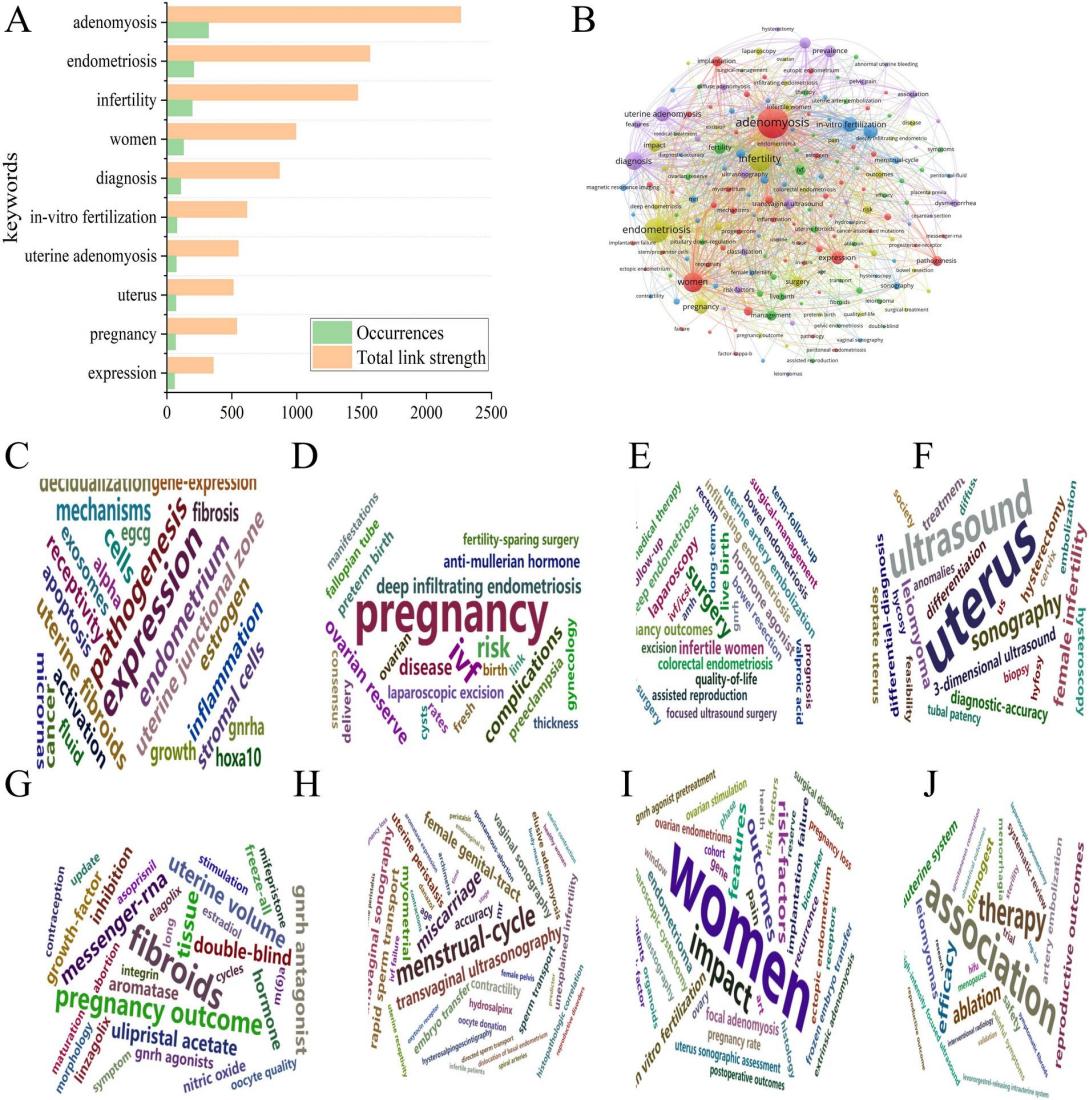
Keywords of the distribution, co-occurrence network diagram, and word cloud cluster map. **(A)** The distribution of keywords: the green histogram represents occurrences, and the orange histogram shows the total link strength. **(B)** The co-occurrence network of keywords; the minimum frequency of occurrences of keywords ≥5. Node size and color represent the frequency of keywords and clusters, respectively. Lines of different colors show that the two keywords appear in an article. **(C–J)** The word cloud cluster map of pathology and mechanisms, adverse pregnancy-associated factors, surgical treatment, diagnosis, assisted reproduction treatment, infertility factors, quality of life, and medical treatment.

**Figure 9:**
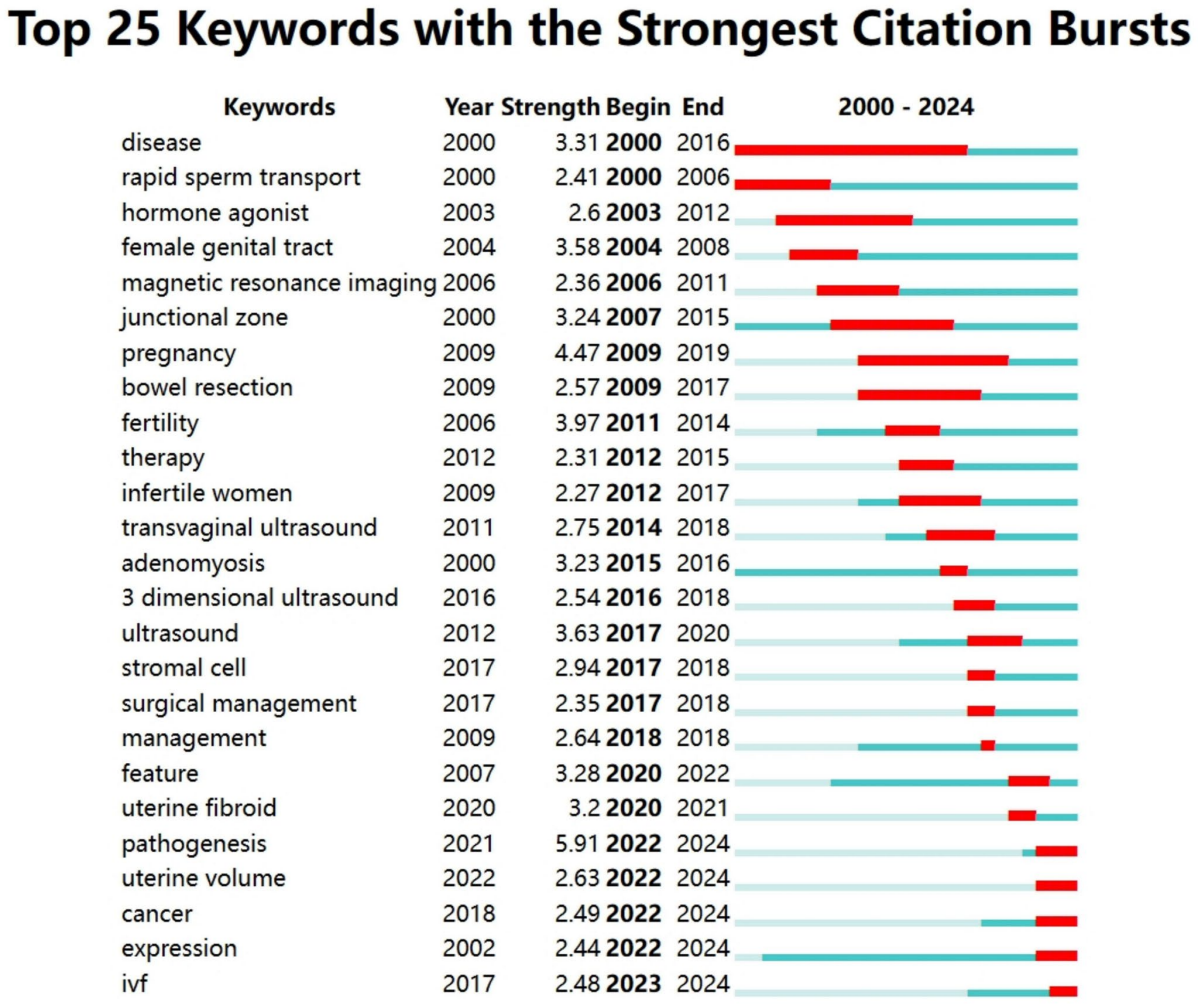
Top 25 keywords with the most vigorous citation bursts. The blue line indicates the timeline, and the red sections indicate the burst duration, including the start and end years.

This analysis included a total of 190 keywords with a frequency exceeding five occurrences. We performed a keyword co-occurrence analysis to further explore hot topics using the Bibliometrix package. Figure 8A display the 10 most common keywords with the strongest associations within the keyword network. The most frequently occurring keywords was AM (*n* = 320; total link strength = 2,263), followed by endometriosis (*n* = 205; total link strength = 1,563), infertility (*n* = 193; total link strength = 1,469), and women (*n* = 127; a total link strength = 993), diagnosis (*n* = 105; a total link strength = 865). High-frequency keywords are valuable for aiding researchers in effectively identifying current hot topics in the field. A network diagram illustrating the most frequently used keywords is shown in Figure 8B. This study identified 190 keywords classified into eight clusters: pathology and mechanisms, adverse pregnancy-associated, surgery treatment, diagnosis, ART, infertility factors, quality of life, and medical treatment (Figures 8C–J). Additionally, we analyzed citation bursts using CiteSpace and displayed the top 25 keywords exhibiting the most significant citation bursts in Figure 9. This figure shows the period during which keyword citation bursts occurred, particularly relating to the disease concepts. For example, disease, rapid sperm transport, and hormone agonists were among the earliest to exhibit citation bursts.

Keywords related to AM treatment and clinical research, including magnetic resonance imaging, junctional zone, pregnancy, bowel resection, infertile women, and transvaginal ultrasound, typically experienced citation burst between 2006 and 2009, with moderate intensity. Notably, our analysis identified keywords that continue to exhibit significant citation burst projected through 2024, including pathogenesis (strength = 5.91; period = 2022–2024), uterine volume (strength = 2.63, period = 2022–2024), cancer (strength = 2.49, period = 2022–2024). These keywords may represent key focal points and objectives in current AM-associated infertility research.

### 3.7 Clustering analysis of keywords

We manually classified the keywords from network data into eight clusters to elucidated the current research trends related to AM-associated infertility. These clusters encompass pathogenesis, adverse factors affecting pregnancy, treatment and diagnostic methods, disease progression, IVF, infertility women, and fertility management.

**Figure 10:**
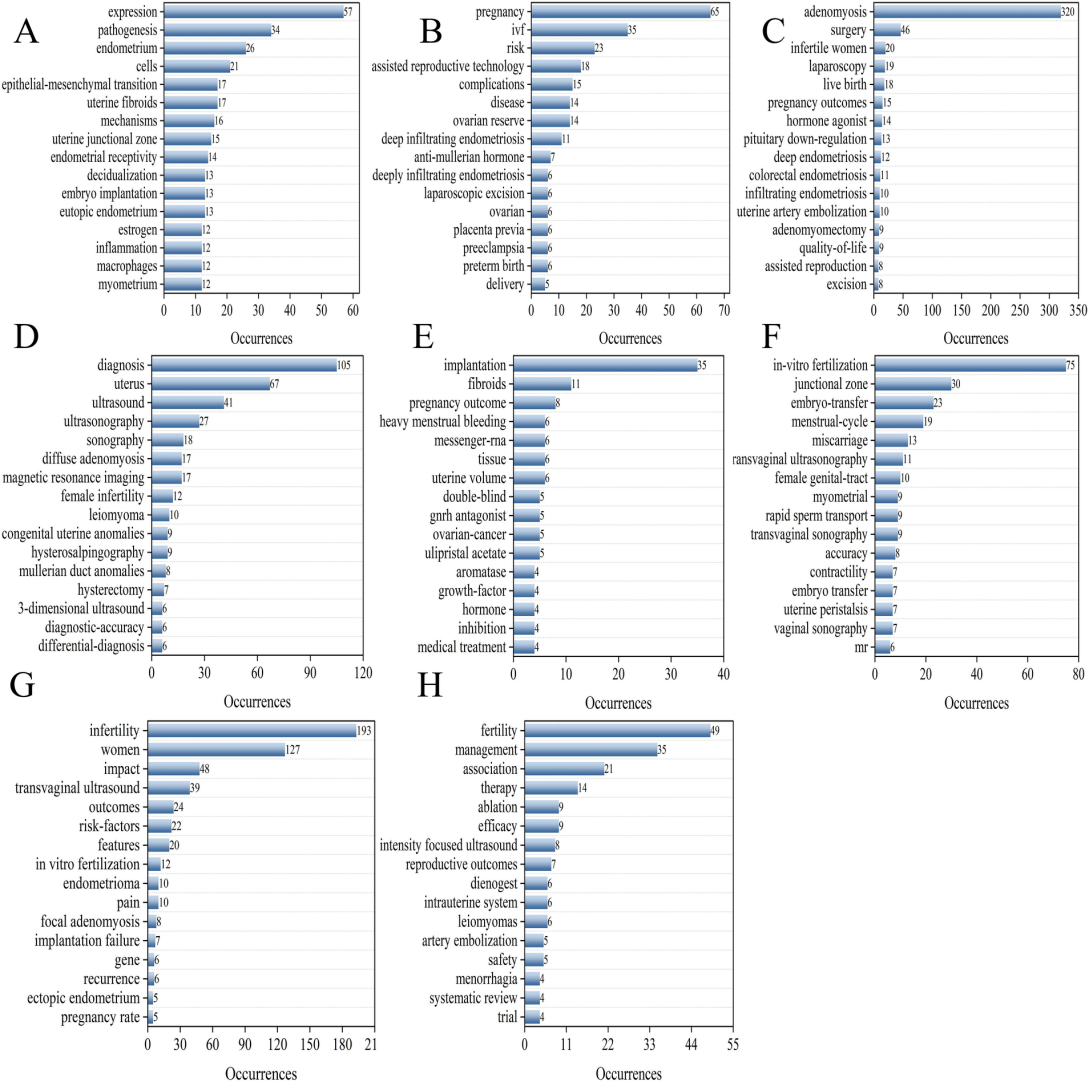
Manual analysis and clustering of keywords. The blue histograms represent the frequency of keyword occurrences. **(A)** Pathogenesis. **(B)** Adverse factors affecting pregnancy. **(C)** Treatment means. **(D)** Diagnosis methods. **(E)** Disease progressive progress. **(F)** *In-vitro* fertilization management. **(G)** Infertility women. **(H)** Fertility management, respectively.

Cluster 1 indicates that AM encompass a spectrum of diseases influenced by epithelial-mesenchymal transition, eutopic endometrium, and inflammation, which collectively impair endometrial receptivity and may cause infertility. Notably, research hotspots in this field predominantly focus on gene expression (Figure 10A). Cluster 2 highlights that AM poses significant risks for pregnancy, resulting in increased complications such as placenta previa, preterm birth, and preeclampsia (Figure 10B). In cluster 3, the primary treatment means for AM have been divided into surgical interventions, hormone therapy, and uterine artery embolization, all of which can adversely affect quality of life of AM patients (Figure 10C). Cluster 4 reveals that the diagnosis of AM primarily relies on ultrasound and magnetic resonance imaging (MRI), but variability in diagnostic methods impacts the accuracy of the results (Figure 10D). From cluster 5, we infer that AM significant impacts the implantation success rates for patients attempting to conceive, as disease progression can lead to myometrial fibrosis and alterations in uterine volume (Figure 10E).

Furthermore, according to cluster 6, AM may increase the difficulty and risk of miscarriage among patients undergoing IVF, possibly lead to damage to the junctional zone. Thus, enhanced pretreatment strategies and vigilant monitoring are essential (Figure 10F). Cluster 7 reinforces that AM is a significant risk factor for infertile women, emphasizing gene-associated pathogenesis, which holds the potential to is expected to address the current challenges of AM-associated infertility effectively (Figure 10G). Lastly, cluster 8 reveals that ensuring fertility preservation in AM patients represents a crucial and challenging long-term objective, necessitating individualized treatment choices such as dienogest, the levonorgestrel-releasing intrauterine system, high-intensity focused ultrasound, or laparoscopic myomectomy (Figure 10H).

## 4 Discussion

This study represents the first analysis of the global research landscape surrounding AM-associated infertility utilizing bibliometrics methodologies. Both AM and infertility are common gynecologic diseases that not only pose significant challenges for individuals but also impose substantial economic burdens on the national healthcare systems, society, and families (29, 30). The reported prevalence of AM can be as high as 70% (31), and it affects approximately 24.4% of infertile women (32). AM is widely recognized for its detrimental affects on fertility, contributing to infertility among women of childbearing age (33–35). Moreover, the incidence of AM associated with infertility is increasing annually, correlating with the tendency of delayed childbearing among women (5, 31). Cozzolino et al. (36) confirmed that women with AM have reduced live birth rates (LBR) (OR 0.59, 95% CI 0.37–0.92, *p* = 0.02), clinical pregnancy rate (OR 0.66, 95% CI 0.48–0.90), and ongoing pregnancy rate (OR 0.43, 95% CI 0.21–0.88), alongside an increased miscarriage rate (OR 2.11, 95% CI 1.33–3.33). Younes et al. (37) reported a 41% reduction in LBR among patients with AM. Vercellini et al. (38) elaborated a 28% decrease in the likelihood of clinical pregnancy via ART compared to women without AM. Additionally, Marvelous et al. (39) indicated a decline in clinical pregnancy among AM patients, ranging from 42.7% with an AM score of zero to 13% with a score of seven.

Consequently, a growing number of scholars are focusing on the relationship between AM and infertility, resulting in a large aggregation of articles and reviews exploring the complex mechanisms underlying this association. Despite this, a systematic method for analyzing and identifying key areas of interest in this research domain has been lacking. Bibliometric analysis, similar to epidemiological approaches, offers a robust means of highlighting potential future research directions by examining authorship, institutional contributions, journal impact, and keyword usage in existing literature. This approach provides valuable insights that can inform and deepen future investigations in the field (10, 40, 41).

In this study, we performed a scientometric analysis to grasp the current research hotspots, keywords, focal points, challenges, and trends pertaining to AM-associated infertility. Our analysis encompassed 456 articles and reviews published across 153 journals by 123 institutions in 51 countries/regions, yielding a total of 13,426 citations and 62 *H*-indexes. We established that AM remains a primary concern and a significant challenge within infertility research globally, with a continuous growth in the quantity of published articles since 2006. The significance of studying the association between AM and infertility is gradually gaining recognition within both academic and clinical circles. Major contributions were identified from China, the USA, Italy, France, and Japan, collectively ranking as the top five countries in terms of publications. This trend may be attributed to the high prevalence of AM and the relatively advanced status of infertility and biomedical study in these nations. We divided 190 keywords, which appeared more than five times, into eight clusters, mainly focused on pathogenesis, adverse factors affecting pregnancy, treatment methods, diagnostic modalities, disease progression, IVF management, infertility, and fertility management. These clusters indicate significant interest in AM-related infertility research over the past 24 years.

Additionally, by analyzing the citation burst of keywords, we discovered emerging research hotspots in the potential mechanisms of pathogenesis, diagnostic methods, and strategies for improving pregnancy success in AM-associated infertility. Recent investigations have increasingly aimed at decoding the intricate mechanism involved in the application of targeted therapies for AM patients with infertility. Although an unambiguous understanding of the pathogenesis is still pending, several hypotheses have gained traction. Altered endometrial function and receptivity in AM patients may give rise to a pro-inflammatory environment and heightened oxidative stress, negatively affecting embryo implantation and survival (42, 43). Other proposed mechanisms include abnormal uterotubal transport caused by adenomyomas with obstruction, which may block sperm transport by distorting the uterine cavity and disrupting normal myometrium structure and function (31, 33). Additionally, irregular uterine contractions during the follicular phase and disturbance in the uterine junctional zone have been implicated as potential contributors to AM-associated infertility (44–46). Recent findings show that increased amplitude and decreased contraction coordination in AM patients could significantly lead to infertility, particularly during the luteal phase when implantation occurs (47). The importance of uterine peristalsis during the peri-implantation phase is notably highlighted in the context of IVF implantation failures and adverse pregnancy outcomes in AM (48). These findings indicate quantifying abnormal patterns and measures of uterine contractility offers a potential new tool for explaining infertility associated with AM (47).

Moreover, evidence of AM with infertility, involving various biomarkers such as matrix metalloproteinases (MMP), interleukins (IL-6, IL-10), HOXA10, leukemia inhibitory factor (LIF), cytochrome P450, and RCAS1 (49). Previous studies suggest that the downregulation of HOXA10, NR4A receptor, and FOXO1A appears to impaired implantation in women with AM (50, 51), and dysregulation of LIF has a similar effect (52). Additionally, molecules like nitric oxide, which are expressed at abnormally high levels, adversely impact sperm transport, implantation, and decidualization, leading to AM-related infertility (53). Pro-oxidative and antioxidative cytokines, including copper (Cu), manganese superoxide dismutase (Mn-SOD), and zinc superoxide dismutase (Zn-SOD), are associated with increased inflammatory responses in the endometrium (54, 55). Consequently, prior research indicates significant opportunities for further investigations into the correlation between AM and infertility.

Although histopathological reports are considered the gold standard for diagnosing AM, they can lead to diagnostic delays of up to 12 years (56) and are not essential for treating patients with concurrent infertility. Instead, imaging techniques serve as the primary diagnostic tools (5, 29). Some studies utilize trans-vaginal ultrasound, while others employ MRI or a combination of both approaches, leading to potential inconsistencies in diagnostic effectiveness (57, 58). The incidence of infertility linked to AM appears to be classification-dependent (59). Moreover, underdiagnosis by less experienced practitioners cannot be discounted, as this may lead to the erroneous inclusion of women with AM in control group, thereby potentially underestimating the actual effect of AM on reproductive outcomes (34). Therefore, the accuracy of diagnosing AM in the context of infertility remains contentious. Further research is imperative to establish uniform diagnostic criteria that clarify the definitive connection between AM and infertility.

Regarding treatment options to improve pregnancy outcomes, there are currently no harmonized international guidelines for managing patients with AM who wish to preserve fertility (60, 61). Nonetheless, available evidence suggests that treatment can positively effect on fertility outcomes (33). For instance, surgical interventions have been shown to increase rates of natural conception (36). Additionally, the use of danazol-loaded devices yield a pregnancy rate of 41%, while GnRHa therapy results in a LBR of 36.2%. Uterine artery embolization has an even higher LBR of 83.3% (35). Other studies report pregnancy rates of 60.5% following complete excision and 46.9% after partial excision of AM (62). The odds ratio of clinical pregnancy post-surgery is reported as 6.22 (CI 2.34–16.54) (37). Furthermore, variations in AM types demonstrate different effects on fertility outcomes, focal AM is associated with a pregnancy rate of 49.1%, compared to 38.5% for the diffuse AM, and a miscarriage rate of 27.6% for focal AM versus 16.2% for the diffuse AM (63).

However, Mijatovic et al. (64), noted no significant increase in clinical pregnancy rates among infertile women with AM who had previously undergone GnRH treatment (36). The overall effectiveness of surgical treatment for AM affecting pregnancy rate remains inconclusive, with a reported risk of uterine rupture estimated at 6.0% (65). A systematic review further indicated that treatments involving oral contraceptives, antiprostaglandins, progestins, danazol, and GnRHa have not improved pregnancy rates for women with AM planning to conceive. However, high-intensity focused ultrasound and combination therapies before ART may benefit these patients (5). Although existing research confirms that pharmacological and surgical treatments for AM positively impact reproductive outcomes, including pregnancy rates and LBR, the comparative effectiveness of different treatments and the optimal timing for delaying pregnancy remain unclear. Additionally, limited evidence on the correlation between infertility and the severity and classification of AM may affect pregnancy rate statistics (66). Therefore, developing standardized protocols to address AM-related infertility is crucial, and the efficacy of these therapeutic options must be validated through prospective randomized controlled trials.

## 5 Limitations and superiority

To the best of our knowledge, our study is the first in-depth scientometric analysis of AM-associated infertility. However, several limitations need attention. First, the data were sourced solely from the SCI-E database within the WoSCC, potentially omitting relevant literature and causing a bias in research conclusions. Second, the use of bibliometric software for author analysis does not currently allow for the differentiation of author name abbreviations, which may lead to inaccuracies. Additionally, bibliometric analysis based on machine algorithms does not permit an in-depth exploration of individual studies, possibly omitting some information. Moreover, as the review focuses exclusively on studies addressing infertility in AM, there may be a selection bias present. Finally, the lack of authoritative guidelines for bibliometric analyses in medical research is a significant challenge for academics who wish to gain a comprehensive and accessible understanding of bibliometric methods and their application in medical research.

However, the WoS is the most powerful search engine, and the WoSCC database contains extensive data on the theme of AM-associated infertility. In addition, the WoS is the premier research platform for biomedical and natural science, and the world’s most trusted publisher with an independent global citation database. Therefore, based on an adequate amount of data and the correct scientometrics methods, the outcomes of this study are convincing and may help accurately identify knowledge gaps, research hotspots, and development trends in AM-associated infertility. The perspectives presented here can guide the generation of novel ideas for further in-depth investigations into AM-associated infertility. Specifically, research on improving uterine receptivity during the peri-implantation period offers direction and encourages further exploration for focused collaboration between researchers and clinicians.

## 6 Conclusion

This study is the first to use bibliometric methods to detail global trends and the current status of AM-associated infertility over the past 20 years. The research highlights that international interest in this complex field remains strong. Key topics include pathogenesis, factors affecting pregnancy, treatment and diagnostic methods, disease progression, and IVF management. Although chronic disease management strategies, pharmacological treatments, and ART have improved fertility outcomes in AM patients, further collaboration between researchers and clinicians is crucial to facilitate translational clinical research. This study aids in identifying research hotspots and fostering regional collaboration for a deeper understanding of the AM-associated infertility landscape and its evolution.

## Data Availability

The original contributions presented in the study are included in the article/supplementary material, further inquiries can be directed to the corresponding authors.

## Footnotes

^1^ www.bibliometrix.org/home/
